# Integrated Multi-Omics Reveals New Ruminal Microbial Features Associated with Peanut Vine Efficiency in Dairy Cattle

**DOI:** 10.3390/life14070802

**Published:** 2024-06-26

**Authors:** Zhanwei Teng, Ningning Zhang, Lijie Zhang, Liyang Zhang, Shenhe Liu, Tong Fu, Qinghua Wang, Tengyun Gao

**Affiliations:** 1 College of Animal Science and Veterinary Medicine, Henan Institute of Science and Technology, Xinxiang 453003, China; tengzhanwei@hist.edu.com (Z.T.);; 2 College of Animal Science and Technology, Henan Agricultural University, Zhengzhou 450046, China; 3 Postdoctoral Research Base, Henan Institute of Science and Technology, Xinxiang 453003, China

**Keywords:** Holstein cow, in situ, peanut vine, rumen degradability, rumen microbiome

## Abstract

**Simple Summary:**

Peanut vines are abundant and cheap, and are a key forage source for ruminants; however, knowledge of the bacterial species that colonize peanut vines over time during rumen incubation remains limited. In this study, in situ nylon bag degradation, scanning electron microscopy, and 16S rRNA gene sequencing and metagenomics technology were used to study the attachment characteristics and function of microorganisms involved in peanut vine degradation, with the aim of providing basic knowledge regarding the utilization of peanut vine and other roughage. Our results demonstrate that the colonization of peanut vine by microorganisms is dynamic in the rumen. The dominant phyla and genera, as well as the structure and function, of the microbial communities varied over time. This experiment identified the key microorganisms and CAZymes were important for plant fiber degradation. Therefore, our data proved new insights into biomass conversion, reveal that numerous bacteria and enzymes are involved in forage degradation in the rumen, and lay the foundation for targeted improvement of crop residue utilization for ruminants.

**Abstract:**

The aim of this study was to improve the utilization of peanut vines as forage material for ruminants by investigating the degradation pattern of peanut vines in the dairy cow rumen. Samples of peanut vine incubated in cow rumens were collected at various time points. Bacterial diversity was investigated by scanning electron microscopy (SEM) and 16S rRNA gene sequencing. Carbohydrate-active enzymes (CAZymes) were analyzed by metagenomics. The peanut vines degraded rapidly from 2 to 24 h, before slowing from 24 to 72 h. SEM images confirmed dynamic peanut vine colonization. Firmicutes and Bacteroidetes were the two most dominant bacterial phyla throughout. Principal coordinates analysis indicated significant microbial composition changes at 6 and 24 h. This may be because, in the early stage, soluble carbohydrates that are easily degradable were degraded, while in the later stage, fibrous substances that are difficult to degrade were mainly degraded. Glycoside hydrolases (GHs) were the most abundant CAZymes, with peak relative abundance at 6 h (56.7 trans per million, TPM), and reducing at 24 (55.9 TPM) and 72 h (55.3 TPM). Spearman correlation analysis showed that Alistipes_sp._CAG:435, Alistipes_sp._CAG:514, Bacteroides_sp._CAG:1060, Bacteroides_sp._CAG:545, Bacteroides_sp._CAG:709, Bacteroides_sp._CAG:770, bacterium_F082, bacterium_F083, GH29, GH78, and GH92 were important for plant fiber degradation. These findings provide fundamental knowledge about forage degradation in the cow rumen, and will be important for the targeted improvement of ruminant plant biomass utilization efficiency.

## 1. Introduction

Peanut oil is an economically important crop in many countries. In China, the annual planting area of peanut crops is more than 5 million hectares, accounting for 35% of all oil crops [1]. There are numerous by-products of peanut production, including peanut hulls, peanut shells, and peanut vines [2]. With the rapidly growing and urbanizing global population, the worldwide demand for animal-derived food will increase sharply by 2030 [3]. Due to limitations on high quality forage, the Chinese dairy industry suffers from a quality forage bottleneck. Peanut vines are abundant and cheap, and are a key forage source for ruminants and geese [4,5]. The efficient utilization of peanut vines in ruminants both reduces feed cost and is a relatively environmentally friendly disposal route for this cellulosic material. The crude protein (CP) content of peanut vine (dry matter basis, DM) is about 12%; its neutral detergent fiber (NDF) and acid detergent fiber (ADF) are about 50% and 36%, and are more easily digestible than alfalfa [6]. A diet combining 45% peanut vine and 55% whole-plant corn silage in the roughage improved the growth performance and meat quality of beef cattle [7]. However, knowledge of the rumen microbiome that colonizes peanut vines over time during rumen incubation remains limited; therefore, increased knowledge of temporal changes in the structure and function of microbial communities colonizing peanut vines in the cow rumen could help us to develop novel strategies aimed at improving ruminant forage utilization efficiency.

The rumen is an elegant and highly evolved cellulose-digesting system that depends on the metabolic activities of the symbiotic microbial communities residing within it, including bacteria, fungi, protozoa, and archaea [8]. Bacteria are the main microorganisms that colonize the rumen, and have important roles in the fermentation and degradation of forage materials. The attachment of rumen microorganisms to feed is a key step in rumen fermentation and digestion [9,10]. A previous study showed that large numbers of microorganisms were attached to ryegrass after it was placed in the rumen for 5 min [11]. Liu et al. [12] found that the microbial communities that adhere to rice straw and alfalfa changed significantly after they had stayed in the rumen for 6 h. Cheng et al. [13] demonstrated that tightly attached microorganisms made a greater contribution to the fiber degradation process, particularly the degradation of roughage after 6 h; however, most of the microorganisms were not classified. The above studies generally applied 16S rRNA gene sequencing to examine the dynamic changes in bacterial communities attached to roughage; however, this approach cannot directly identify the functions of the microbes under study. In contrast, metagenomics can reveal the identities and potential functions of microorganisms [14,15]. Therefore, a combination of 16S rRNA analysis and metagenomic sequencing can enable a clearer elucidation of the complexities of microbial community structure and function during the process of roughage degradation.

Recent studies have demonstrated that the physical and chemical properties of feed influence colonization by rumen microbes. Fibrobacteres were significantly overrepresented in bacterial communities attached to forage with a high neutral detergent fiber (NDF) content, while *Ruminococcus* tended to attach to forage with a low acid detergent lignin (ADL) content [10], demonstrating that the type of forage is a key factor that affects the degradation and microorganism attachment processes in the rumen. Therefore, understanding the temporal and spatial programmatic changes in the structure and function of microbial communities colonizing peanut vine in the rumen is an important prerequisite for improving peanut vine utilization. In this study, we designed and conducted in situ nylon bag degradation, scanning electron microscopy (SEM), 16S rRNA, and metagenomic experiments to study the degradation pattern of peanut vine and identify the changes and functions of bacteria attached to peanut vine during rumen degradation.

## 2. Materials and Methods

### 2.1. In Situ Rumen Incubation and Forage Sample Collection

All procedures were approved by the Animal Care and Use Committee of Henan Agricultural University (approval number: HENAU-2018-015). Three rumen-cannulated Holstein cows of similar age (3.5 to 4 years) were used in this experiment. Cows were housed in individual stalls, with free access to water. Diet was fed as total mixed ratio, twice daily (at 06:00 and 17:00 h). The detailed ingredient composition of the diet (dry matter (DM) basis) was 45% concentrate supplement, including gluten 10%, corn 34%, Soybean meal 15%, DDGS 15%, bran 15%, Cottonseed meal 5%, sesame meal 5%, premix 1% (Hefeng Feed Co., Ltd., Jiaozuo, China), 25% corn silage, 15% alfalfa hay, and 15% peanut vine.

Peanut vines were collected in September from a peanut vine field in Henan, China. Peanut vines were dried at 65 °C for 72 h, then crushed through a 0.5 mm sieve to generate peanut vine powder, which was packaged and stored in sealed plastic bags at room temperature before use. The chemical composition of peanut vine (DM basis) is CP 9.16%, NDF 49.03%, ADF 36.03%, crude fat 2.68%, crude ash 7.90%. In the experiment, peanut vine samples (approximately 5 g) were weighed into individual nylon bags (8 × 12 cm, 50 μm pore size), the sample mass-to-bag surface area ratio was 0.05 g/cm^2^. Before morning feeding, eighty-four nylon bags were prepared and placed into the rumens of three cannulated Holstein cows (28 bags per cow). A total of twelve bags (4 bags per cow) for each time point were retrieved from the rumens of the three cows at 0.5, 2, 6, 12, 24, 48, and 72 h and washed gently with phosphate-buffered saline (PBS, pH 7.4) to remove the rumen contents on the outer surface of the bag. At 0 h, nylon bags were not placed in the rumen for measuring soluble components.

The upper two nylon bags were washed with running tap water until the water ran clear, and were then dried at 65 °C for analysis of the relative degradation of peanut vine biomass. Two nylon bags in the lower area were used to collect bacterial community samples for SEM analysis. SEM samples were collected at 0 h (before fermentation) and at each sampling time point. A few residue fragments were removed from each bag and fixed with 2.5% glutaraldehyde for SEM. Residue samples (1 g) were used to isolate adherent fractions, as described previously [16]. These fractions were placed in liquid nitrogen and subsequently transferred to the laboratory and stored at −80 °C until analysis of bacterial community and carbohydrate active enzyme (CAZyme) profiles.

### 2.2. Biomass Degradation Analysis

Relative peanut vine biomass degradation was determined by DM, NDF, and ADF analysis. The DM content of the peanut vine was determined according to methods described by the Association of Official Analytical Chemists [17], and the NDF and ADF content according to Van Soest et al. [18].

The rumen degradation parameters were calculated based on the formula proposed by Orskov et al. [19]: Y = a + b × (1 − e^−ct^), where Y represents the degradation rate of feed components at time t, A is the rapid degradation part of feed, B is the slow degradation part of feed, C is the degradation rate constant of the slow degradation part, and T is the residence time of the feed sample in the rumen.

### 2.3. Scanning Electron Microscopy

Alteration of the physical structure of peanut vine was visually examined by SEM [20]. Briefly, samples were fixed for >24 h at 4 °C, washed three times with PBS for 10 min each, and then dehydrated in 50%, 70%, 80%, and 90% ethanol (15 min each) and 100% ethanol three times (30 min each). The ethanol was then replaced with tertiary butyl alcohol before critical point drying using a Hitachi freeze dryer (ES-2030, Hitachi, Tokyo, Japan). Each dried sample was then sputter coated with approximately 10 nm of Au/Pd using a Hitachi E-1010 Ion sputter instrument (Hitachi, Tokyo, Japan). SEM imaging was conducted using a Hitachi SU-8020 microscope (Hitachi, Tokyo, Japan).

### 2.4. Analysis of Bacteria Attached to Peanut Vine by Illumina MiSeq Sequencing of 16S rRNA Genes

Microbial community genomic DNA was extracted from peanut vine samples using the E.Z.N.A.^®^ soil DNA Kit (Omega Bio-tek, Norcross, GA, USA), following the manufacturer’s protocols. The integrity of extracted DNA was checked by 1% agarose gel electrophoresis, and DNA concentration and purity were measured using a NanoDrop 2000 UV-vis spectrophotometer (Thermo Scientific, Wilmington, DE, USA). The V3–V4 hypervariable region of the 16S rRNA gene was amplified by PCR using the following primer pairs: 338F (5′-ACTCCTACGGGAGGCAGCAG-3′) and 806R (5′-GGACTACHVGGGTWTCTAAT-3′) [21]. The integrity of PCR products was verified by 2% agarose gel electrophoresis and the products were purified using the AxyPrep DNA Gel Extraction Kit (Axygen Biosciences, Union City, CA, USA). Purified amplicons were pooled in equimolar amounts and paired-end (PE) sequencing (2 × 300 bp) was conducted using an Illumina MiSeq platform (Illumina, San Diego, CA, USA), according to standard protocols from Majorbio Bio-Pharm Technology Co., Ltd. (Shanghai, China).

The resulting sequences were demultiplexed, quality-filtered using Trimmomatic and merged using FLASH(version1.2.7). The exaction criteria were as follows: (i) 300 bp reads were truncated at any site receiving an average quality score < 20 over a 50 bp sliding window; (ii) truncated reads < 50 bp and reads containing ambiguous characters were also discarded; (iii) sequences with overlap > 10 bp were assembled according to their overlapped sequence; (iv) barcodes were matched exactly, allowing for a two nucleotide mismatch in primer matching. Chimeric sequences were identified and removed using UCHIME(version4.2). Subsequently, operational taxonomic units (OTUs) were clustered using UPARSE (version 7.1, http://drive5.com/uparse/, accessed on 1 December 2020) with a 97% similarity cutoff. The taxonomy of each OTU representative sequence was performed using Ribosomal Database Project Classifier (http://rdp.cme.msu.edu/, accessed on 1 December 2020) by comparison against the Silva (SSU128)16S rRNA database (confidence cutoff, 0.7).

### 2.5. Bacterial Community and CAZyme Analysis of Bacteria Attached to Peanut Vine by Metagenome Shotgun Sequencing

#### 2.5.1. DNA Extraction, Library Construction, and Metagenomic Sequencing

Total DNA extraction and quality control were carried out as described for 16S analysis. DNA samples were fragmented to an average size of approximately 400 bp using a Covaris M220 instrument (Gene Company Limited, Shanghai, China) for PE library construction. PE libraries were constructed using NEXTFLEX^®^ Rapid DNA-Seq (Bioo Scientific, Austin, TX, USA). Qualified libraries were analyzed on the Illumina NovaSeq (Illumina Inc., San Diego, CA, USA) platform at Majorbio Bio-Pharm Technology Co., Ltd. (Shanghai, China).

#### 2.5.2. Sequence Quality Control and Genome Assembly

Adapter sequences were stripped from the 3′ and 5′ ends of PE Illumina reads using SeqPrep (https://github.com/jstjohn/SeqPrep, accessed on 1 December 2020). Low-quality reads (length < 50 bp, quality value < 20, or containing N bases) were removed using Sickle (https://github.com/najoshi/sickle, accessed on 1 December 2020). Reads were aligned to the cattle genome using BWA (http://bio-bwa.sourceforge.net, accessed on 1 December 2020), and any hits associated with the reads and their mated reads were removed. Metagenomics data were assembled with MEGAHIT (https://github.com/voutcn/megahit, accessed on 2 December 2020), which uses succinct de Bruijn graphs [22]. Contigs of ≥300 bp were selected for inclusion in the final assembly, and contigs used for further gene prediction and annotation.

#### 2.5.3. Gene Prediction, Taxonomy, and Functional Annotation

Open reading frames (ORFs) from each assembled contig were predicted using MetaGene (http://metagene.cb.k.u-tokyo.ac.jp/, accessed on 3 December 2020) [23]. Predicted ORFs ≥ 100 bp were retrieved and translated into amino acid sequences using the NCBI translation table (http://www.ncbi.nlm.nih.gov/Taxonomy/taxonomyhome.html/index.cgi?chapter=tgencodes#SG1, accessed on 3 December 2020).

All predicted genes with ≥95% sequence identity (≥90% coverage) were clustered using CD-HIT (http://www.bioinformatics.org/cd-hit/, accessed on 4 December 2020), and the longest sequences from each cluster were selected as representatives for the construction of a non-redundant gene catalog [24]. After quality control, reads were mapped to representative sequences with 95% identity using SOAP aligner (http://soap.genomics.org.cn/, accessed on 4 December 2020), and the gene abundance in each sample was evaluated [25].

Representative non-redundant gene sequences were aligned to the NCBI NR database with an e-value cutoff of 1 × 10^−5^, using BLASTP (Version 2.2.28+, http://blast.ncbi.nlm.nih.gov/Blast.cgi, accessed on 5 December 2020), for taxonomic annotation [22]. CAZymes annotation was conducted using hmmscan (http://hmmer.janelia.org/search/hmmscan, accessed on 6 December 2020), by screening against the CAZy database Version 5.0 (http://www.cazy.org/, accessed on 6 December 2020), with an e-value cutoff of 1 × 10^−5^. 

### 2.6. Statistical Analysis

Community richness and diversity of samples were calculated using the Shannon, Simpson, Chao1, and ACE indices [26]. The statistical significance of differences between groups was tested by one-way ANOVA. Beta diversity was calculated using QIIME. Beta diversity was evaluated by principal coordinates analysis (PCoA) and used to evaluate differences in species complexity among samples.

Statistical analyses of biomass degradation data (DM, NDF, ADF), and relative abundance of bacterial and CAZyme gene families, were conducted by one-way ANOVA using SPSS 18.0 (IBM, New York, NY, USA). Duncan’s test was used to compare differences between means. Differences are reported as significant at *p* < 0.05; *p* < 0.01 was considered highly significant, and 0.05 ≤ *p* < 0.10 was designated as a tendency toward significance.

## 3. Results

### 3.1. Degradation of Peanut Vine

**Figure 1:**
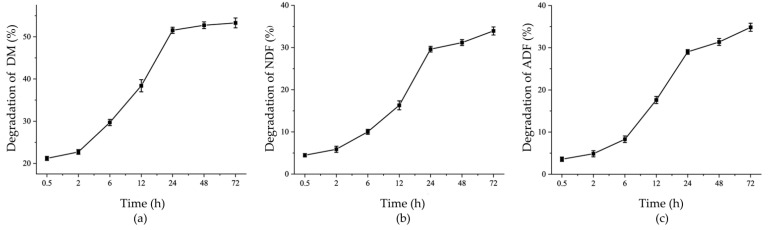
Peanut vine degradation during in situ rumen incubation. DM (dry matter, (**a**) NDF (neutral detergent fiber, (**b**), ADF (acid detergent fiber, (**c**). Error bars represent standard error of the mean.

As shown in Figure 1 and Table 1, the DM degradation rate of the peanut vine was 21.18% within 0.5 h, the peanut vine was rapidly degraded between 6 and 24 h, and DM degradation was almost complete at 24 h. NDF and ADF digestibility showed similar trends to that of DM.

### 3.2. Scanning Electron Microscopy

**Figure 2:**
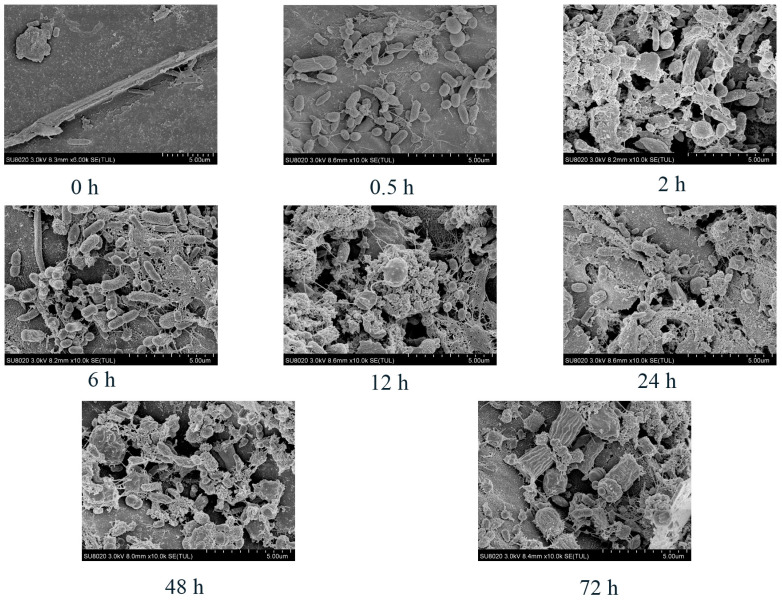
Scanning electron micrographs showing the dynamics of peanut vine degradation and microorganism colonization on peanut vine during in situ rumen incubation.

Large numbers of rumen microorganisms were detected on the peanut vine within 0.5 h after incubation. At 6 h, the number of microorganisms increased and it can be seen that the roughage had already been degraded into fragments. After 24 h, an increased number of microorganisms colonizing the peanut vine and significant degradation fragments of the peanut vine were observed. Until 72 h, the number of microorganisms remained at a high level and peanut vine degradation was visible (Figure 2). The SEM images confirmed the dynamic degradation of peanut vine throughout its incubation in the rumen.

### 3.3. Temporal Changes in Bacterial Communities Attached to Peanut Vine Determined by 16S rRNA Gene Sequencing

**Figure 3:**
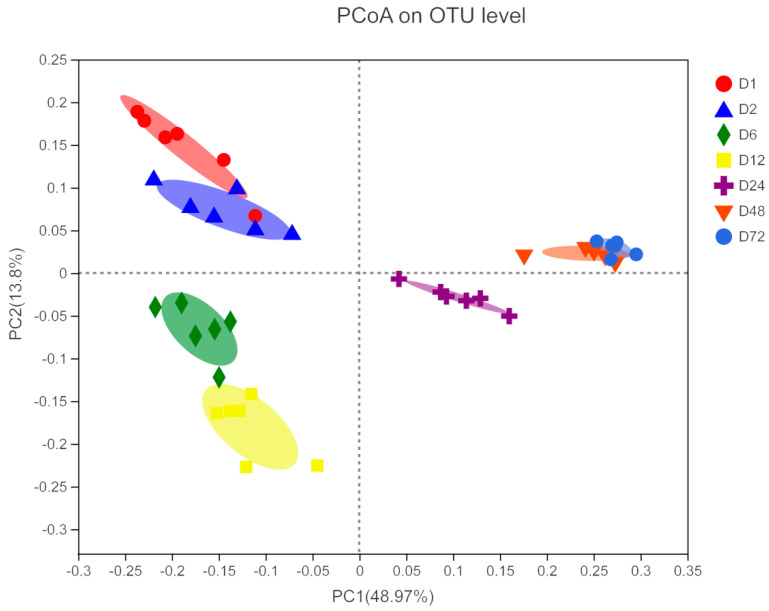
Principal coordinates analysis of bacteria communities attached to peanut vine after incubation for 0.5, 2, 6, 12, 24, 48 and 72 h in cow rumen based on 16S rRNA gene sequencing data. D1, D2, D6, D12, D24, D48, and D72, peanut vine incubation in the cow rumen for 0.5, 2, 6, 12, 24, 48, and 72 h, respectively.

The alpha diversity indices of microorganisms attached to the peanut vine are presented in Table 2. All samples showed high coverage (>97%) at all sampling timepoints, demonstrating that our results determined >97% of the diversity of the microbes attached to the peanut vine. Incubation time significantly affected the Sobs, Shannon, Simpson, Ace, and Chao indices (*p* < 0.05), with the values of the Sobs and Shannon indices being significantly lower at 48 and 72 h, compared with the other time points (*p* < 0.05). This result indicates that bacterial richness and diversity varied according to length of incubation time in the rumen. The beta diversity analysis showed that samples from each timepoint clustered together in separate groups (Figure 3), and that the microbial community structure showed clear shifts at 6 and 24 h. These findings indicate that the rumen microbiota may preferentially attach to forage material at different stages of incubation.

**Figure 4:**
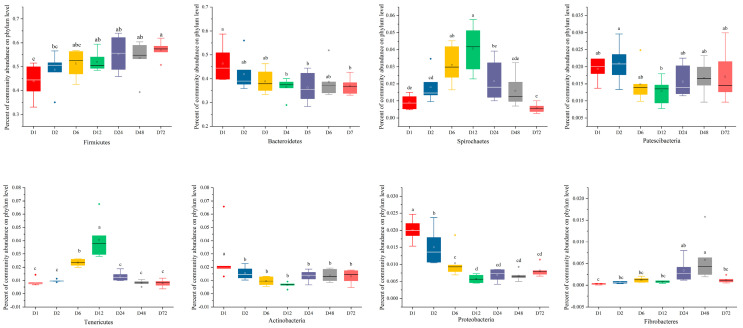
Analysis of microbiota community composition at the phylum level based on 16S rRNA gene sequencing data. D1, D2, D6, D12, D24, D48, and D72 represent peanut vine samples incubated in the cow rumen for 0.5, 2, 6, 12, 24, 48, and 72 h, respectively. Different letters (a–d) indicate significant differences (*p* < 0.05).

Variations in the abundances of major bacterial phyla attached to the peanut vine are presented in Figure 4. Firmicutes and Bacteroidetes were the dominant phyla, accounting for >90% of the total sequences. From 0.5 to 72 h, the relative abundance of Firmicutes increased from 43.89% to 57.04%, and that of Bacteroidetes decreased from 46.33% to 36.86%; however, the total relative abundance of these two phyla combined increased from 90.22% to 93.90%. Compared with 0.5 h, the relative abundance of Firmicutes increased significantly at 24 and 72 h (*P* < 0.05), while the relative abundance of Bacteroidetes decreased significantly at the same time points (*P* < 0.05). These results show that the dominant phyla remained the same at the phylum level, while their relative abundance differed over time.

**Figure 5:**
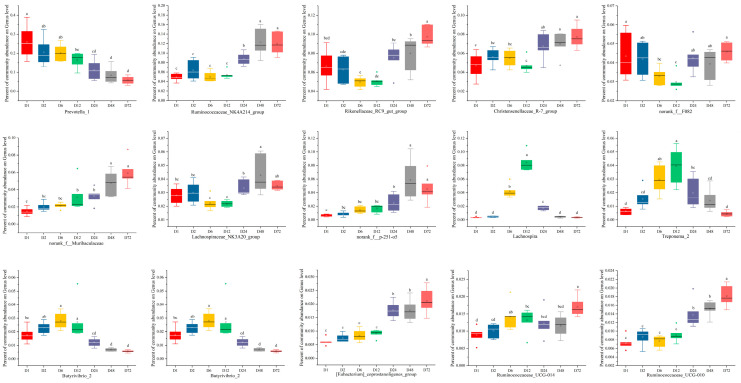
Analysis of microbiota community composition at the genus level based on 16S rRNA gene sequencing data. D1, D2, D6, D12, D24, D48, and D72 represent peanut vine samples incubated in cow rumen for 0.5, 2, 6, 12, 24, 48, and 72 h, respectively. Different letters (a–d) indicate significant differences (*p* < 0.05).

Analysis of microbial community composition focused on genera with relative abundance > 1% at different timepoints during peanut vine degradation is presented in Figure 5. The results show that the main 15 bacterial genera present during the degradation process were as follows: *Prevotella_1*, *Ruminococcaceae_NK4A214_group*, *Rikenellaceae_RC9_gut_group*, *Christensenellacease_R-7__group*, *unclassified_o__Clostridiales*, *norank_f__F082*, *Succiniclasticum*, *norank_f__Muribaculaceae*, *Lachnospiraceae_NK3A20_group, norank_f__p-251-o5*, *Lachnospira*, *Treponema_2*, *Butyrivibrio_2*, *Prevotellaceae_UCG-003*, and *Prevotellaceae_UCG-001*. The relative abundance of *Prevotella_1* decreased, while that of *Ruminococcaceae_NK4A214_group* increased gradually over time during incubation in the rumen.

### 3.4. Temporal Changes in Bacterial Communities Attached to Peanut Vine and CAZymes Determined by Metagenomics

#### 3.4.1. Profiling of the Rumen Metagenome

**Figure 6:**
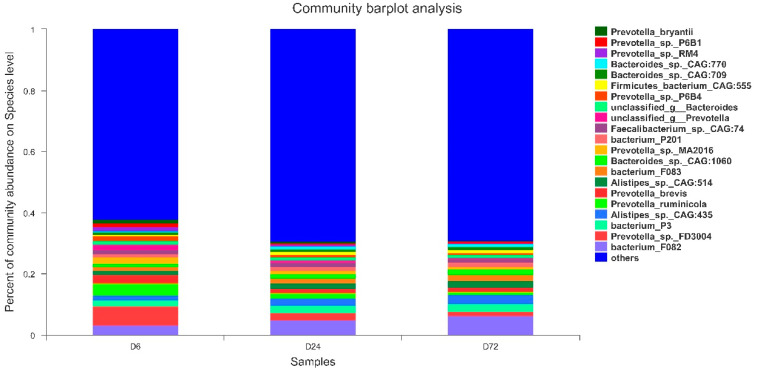
Relative abundance analysis of microbiota communities attached to peanut vine at the species level after incubation for 6, 24, and 72 h in cow rumen based on metagenome shotgun sequencing data. D6, D24, and D72 represent peanut vine samples incubated in cow rumen for 6, 24, and 72 h, respectively. Species with relative abundance < 0.01 in all samples were classified as others.

Based on bacterial diversity and the Bray–Curtis metric, nine samples were selected at 6, 24, and 72 h and used for shotgun metagenome sequencing. A total of 387,202,986 reads were detected, with an average of 43,022,554 ± 685,888 reads per sample. After quality control and removing host genes, a total of 383,600,452 reads were retained (mean, 42,622,272 ± 691,235 reads per sample). In addition to bacteria, metagenomics also detected other microorganisms, including archaea, eukaryotes, and viruses. In this study, organisms with a relative abundance > 1% or ranking in the top 20 were all bacteria; therefore, only the roles of bacteria in the process of peanut vine degradation were investigated. Consistent with the results of the 16S rRNA sequencing, our metagenomics findings also reflected different characteristics of bacteria at the phylum and genus levels. Bacteroidetes and Firmicutes were the dominant phyla (Figure S1), while *Prevotella* was the dominant genus (Figure S2). Microbes present with a relative abundance > 1% at the species level are presented in Figure 6. *Prevotella_sp._FD3004*, *bacterium_F082*, and *bacterium_P3* were the main microbial species present during fiber degradation.

#### 3.4.2. Functional Analysis of Bacteria Attached to Peanut Vine Predicted from Metagenome Shotgun Sequencing Data

PCoA profile of CAZymes at the Class and Family levels using the Bray–Curtis metric indicated differences in diversity values among the 6, 24, and 72 h groups (Figure S3). Further, ANOSIM analysis showed significant dissimilarities between the 6, 24, and 72 h groups (*p* < 0.05). These results demonstrate that the difference between groups was significantly greater than that within groups, and that grouping was representative.

**Figure 7:**
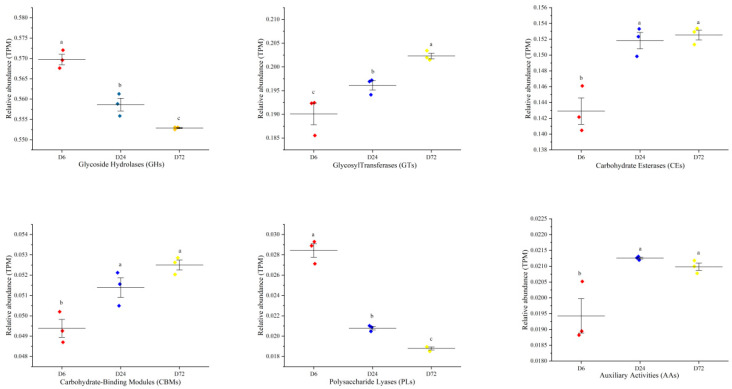
Comparisons of the relative abundance (trans per million, TPM) of CAZyme gene families in bacterial attached to peanut vine at 6, 24, and 72 h. Different letters (a–c) indicate significant differences (*p* < 0.05).

To specifically explore the microbial potential for forage degradation, we screened for CAZymes in the assembled contigs. Among the six classes of CAZyme, unique genes were assigned to 16 distinct families of auxiliary activities (AAs), 61 families of carbohydrate-binding module (CBMs), 16 families of carbohydrate esterases (CEs), 230 families of glycoside hydrolases (GHs), and 73 families of associated glycoside transferases (GTs), as well as 58 families of associated polysaccharide lyases (PLs). As illustrated in Figure 7, the relative abundance of GHs was the highest during the peanut vine degradation process, with the highest relative abundance at 6h, and a downward trend at 24 and 72 h (*p* < 0.05). The relative abundance of PLs was also highest at 6 h, and decreased significantly at 24 and 72 h (*p* < 0.05). In contrast, the relative abundances of GTs, CEs, and CBMs significantly increased with an extended incubation time in the rumen (24 and 72 h), compared with their relative abundance values at 6 h (*p* < 0.05).

**Figure 8:**
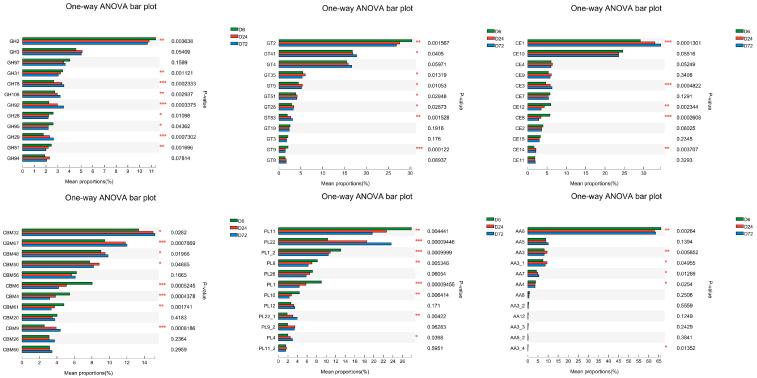
Mean proportions of expression of unigenes annotated to CAZyme families. D6, D24, and D72 represent peanut vine samples incubated in cow rumen for 6, 24, and 72 h, respectively. * 0.01 *< p* ≤ 0.05, ** 0.001 *< p* ≤ 0.01, *** *p* ≤ 0.001.

The mean proportions of the expression of unigenes annotated to CAZyme families are presented in Figure 8. The relative abundances of GH2, GH28, GH31, and GT2 decreased with incubation time in the rumen (*p* < 0.05), while those of CE1, GT4, GT41, GH78, GH92, and GH106 increased (*p* < 0.05). Therefore, the relative abundance of CAZymes changed dynamically at the family level with extended peanut vine incubation time in the rumen.

**Figure 9:**
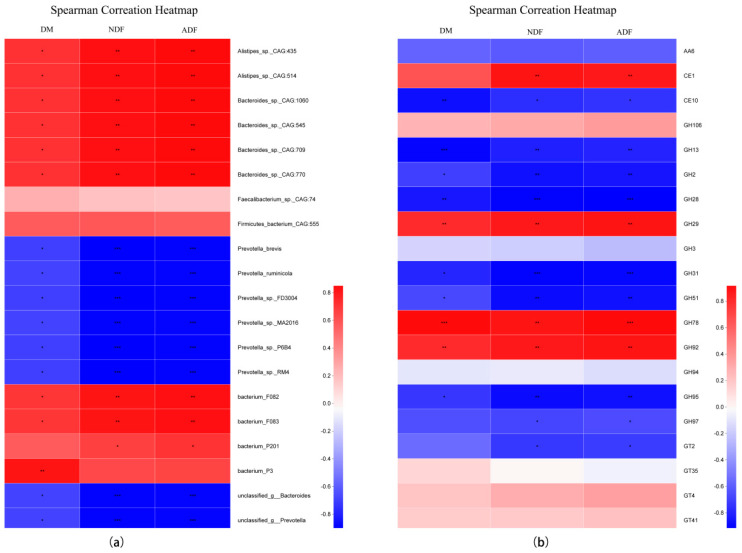
Association analysis. (**a**) Spearman’s correlation analysis between rumen microbiota and biomass degradation rate. (**b**) Spearman’s correlation analysis between CAZyme families and biomass degradation rate. DM, dry matter; NDF, neutral detergent fiber; ADF, acid detergent fiber. R > 0.5 and * 0.01 *< p* ≤ 0.05, ** 0.001 *< p* ≤ 0.01, *** *p *≤ 0.001.

Spearman correlation analysis (Figure 9) showed that Alistipes_sp._CAG:435, Alistipes_sp._CAG:514, Bacteroides_sp._CAG:1060, Bacteroides_sp._CAG:545, Bacteroides_sp._CAG:709, Bacteroides_sp._CAG:770, bacterium_F082, and bacterium_F083 were positively correlated with the DM, NDF, and ADF degradation rates. Further, Bacterium_P201 was positively correlated with the NDF and ADF degradation rates and Bacterium_P3 was positively correlated with DM; however, Prevotella_brevis, Prevotella_ruminicola, Prevotella_sp._FD3004, Prevotella_sp._MA2016, Prevotella_sp._P6B4, Prevotella_sp._RM4, unclassified_g__Bacteroides, and unclassified_g__Prevotella were negatively correlated with the DM, NDF, and ADF degradation rates. The GH29, GH78, and GH92 levels were positively correlated with the DM, NDF, and ADF degradation rates, while the CE1 levels were positively correlated with the NDF and ADF degradation rates. The CE10, GH13, GH2, GH28, GH31, GH51, and GH95 levels were negatively correlated with the DM, NDF, and ADF degradation rates, whereas GH97 and GT2 was negatively correlated with the NDF and ADF degradation rates.

To explore the functional contributions of the identified microbiota, a stacked chart was generated by calculating the relative abundance of microbial taxa relative to their functions (Figure S4). Clear contributions between active taxa (species level) and CAZymes were detected. *Prevotella_sp._FD3004*, *Bacterium_F082*, *Prevotella_ruminicola, Alistipes_sp._CAG:435*, and *Bacterium_F082* were the primary contributors to the GH2, GT2, CE1, CE10, and GT41 families in the attached bacterial community metagenomes. Species and functional regression analyses were used to evaluate the consistency between species and function (Figure S5a). The results showed that there was a significant correlation between microbial community and function (R^2^ = 0.9953, *p* < 0.01). The results of correlation analysis between species and functions showed that some species had correlations with multiple functions (Figure S5b). In this study, *Prevotella_sp._FD3004* was correlated with 13 functions, including a positive correlation with GH95, GH97, GT2, and GH13, and a negative correlation with GH78, GH92, and CE1, demonstrating that a microbe can be associated with one or more functions involved in fiber degradation, and that specific functions can be conducted by multiple microorganisms.

## 4. Discussion

In this study, in situ nylon bag degradation, SEM, and 16S rRNA gene sequencing and metagenomics technology were used to study the attachment characteristics and function of microorganisms involved in peanut vine degradation, with the aim of providing basic knowledge regarding the utilization of peanut vine and other roughage. In situ nylon bag degradation technology is a common method used to evaluate the nutritional value of feed [27]. The DM degradation rate is an important parameter used to evaluate the nutritional value of feed, and is affected by many factors, including the physiological characteristics and nutritional components of feed [10,28] and treatment modes [29,30]. In this study, the peanut vine DM degradation rate was 21.18% in the first 0.5 h, did not differ significantly between 0.5 and 2 h, and was almost completed after 24 h, decreasing thereafter. The DM degradation characteristics of peanut vines are similar to those of ryegrass and rice straw [14,31]. During the first 0.5 h, the peanut vine degraded rapidly, due to the utilization of soluble nutrients in roughage by rumen microorganisms. From 0.5 to 2 h, the peanut vine degradation stagnated temporarily, which may be explained to some extent by the rapid degradation of polysaccharides in the initial stage of fermentation, resulting in an increase in the partial pressure of hydrogen in the rumen inhibiting the degradation of roughage [32,33]. The degradation rate of the peanut vine decreased gradually after 24 h, possibly because the amount of easily degradable substances had been depleted leaving highly lignified tissues that are not readily degraded. Shen et al. [34] found that it was difficult for microorganisms to further degrade and digest rice straw at later stages of fermentation due to the resistance of waxy and siliceous layers in the epidermis. The degradation rate of NDF and ADF are important indices for evaluating the availability of feed, and can reflect the difficulty of feed digestion. The total degradation rate of DM in roughage largely depends on its NDF content [10]. Also, the particle size of substrate could affect the NDF degradation [35]. Mould et al. [36] reported a decreasing influence of this parameter after 96 h. Our results show that the degradation trends of NDF and ADF in peanut vine were similar to that of DM, indicating that NDF and ADF degradation limited that of DM.

Bacteria play key roles in the degradation and fermentation of most feed biopolymers [37]. SEM analysis showed that the peanut vine degradation process was dynamic, consistent with the findings of previous studies [20]. In this study, we found that bacteria attached to peanut vines were primarily Firmicutes and Bacteroidetes at the phylum level, accounting for >90% of total sequences, similar to the dominant bacteria in rumen fluid [38]. According to Cheng et al. [13], Firmicutes and Bacteroidetes are the main microbes attached to roughage, and are involved in the degradation of fiber and polysaccharides [39]. From 0.5 to 72 h, the relative abundance of Firmicutes increased from 43.89% to 57.04%, while that of Bacteroidetes decreased from 46.33% to 36.86%, which is not consistent with the findings of Jin et al. on rice straw [20], likely because rumen microbial communities may have different preferences for attachment to various lignocellulosic feedstuffs [10]. At the genus level, *Prevotella*, *Ruminococcaceae*, *Treponema_2*, *Butyrivibrio_2*, and the *Lachnospiraceae* were the dominant taxa attached to peanut vine. *Prevotella* and *Ruminococcaceae* are established as involved in rumen fiber degradation [40,41]. Liu et al. [12] reported that *Prevotella*, *Ruminococcus*, *Butyrivibrio*, *Fibrobacter*, and *Treponema* were the dominant microbial communities identified during the dynamic attachment process of rumen microorganisms to rice straw. These findings imply that these microorganisms may be crucial for the degradation of fiber; therefore, their functions warrant further investigation.

In this study, PCoA showed that the microbial composition changed significantly at 6 and 24 h, and tended to be stable at 48 and 72 h, which may be closely related to the feed degradation process. In the initial stage of the experiment, rumen microorganisms competed for attachment to the peanut vine; however, when the degradation of easily digestible components was completed, the microbial communities on the surface of the forage materials were relatively uniform [10]. Liu et al. [12] studied the dynamic processes of rumen microorganism attachment to rice straw and alfalfa, and found that the microbes attached to feed changed significantly at around 6 h. Huws et al. [42,43] found that the rumen microbes attached to perennial ryegrass at 0–2 h were significantly different from those after 4 h. The transformation of these microorganisms indicates that they may have different functions in fiber degradation. Therefore, to improve plant fiber utilization, the functions of these microorganisms require clarification.

Metagenomics is the study of the genetic material of entire biological communities, and can be used to obtain information on microbial diversity and function [14]. Consistent with the results of 16S rRNA gene sequencing, the results of the metagenomics analysis in this study demonstrated the differential characteristics of bacteria at phylum and genus levels. At the species level, the majority of microorganisms with relative abundance > 1% belonged to the genus *Prevotella*. *Prevotella* are major rumen microbes that can produce succinate and acetate from starch and protein [44]. With the rapid degradation of easily digestible components, the overall metabolism of roughage is gradually transferred to cellulose lignin metabolism. Therefore, microorganisms also changed from *Prevotella*, which metabolizes soluble carbohydrates, to *Bacillus* and *Treponema_2*, which are involved in the metabolism of fiber and hemicellulose transformation [45]. These results reveal that rumen microbiota exhibit preferences for attachment to forage components, consistent with previous reports [10].

In this study, we found that CAZymes encoded by enriched genes mainly included AAs, CBMs, CEs, GHs, GTs, and PLs. In the six functional classes of CAZymes, GHs and PLs are involved in the cleavage of glycosidic bonds between two sugar units or between a sugar and a non-sugar moiety [46]. The abundances of GHs and PLs were higher in the early stage of the experiment, and subsequently decreased gradually. This was due to the significantly higher levels of GHs and PLs at 6 h than at 24 and 72 h; *Prevotella* can degrade starch and plant cell wall polysaccharides, but not cellulose [34]. AAs are a group of lignin-decomposing enzymes or polysaccharide-decomposing monooxygenases, which act synergistically with other CAZymes [47]. CBMs enable CAZymes to bind to their matrix [48] and likely have an important role in the digestion of crystalline cellulose in plant cell wall biomass [49]. Our results show that the relative abundances of AAs and CBMs increased with roughage incubation time in the rumen. This may be associated with the ability of AAs and CBMs to degrade cellulose, suggesting that the function of microbial communities varied over time. This experiment identified the key CAZymes that were important for plant fiber degradation that can be developed into robust catalytic agents to potentiate the production of biofuels from cellulosic biomass or serve as feed enzymes.

Spearman correlation analysis showed that Alistipes_sp._CAG:435, Alistipes_sp._CAG:514, Bacteroides_sp._CAG:1060, Bacteroides_sp._CAG:545, Bacteroides_sp._CAG:709, Bacteroides_sp._CAG:770, Bacterium_F082, Bacterium_F083, GH29, GH78, and GH92 were positively correlated with the DM, NDF, and ADF degradation rates. Previous studies suggest that rumen bacteria are positively correlated with DM [8]. Wang et al. [50] reported that Alistipes was the primary contributor from the oligosaccharide-degrading enzymes and that GH29, GH78, GH92 were associated with cellulolytic functions. The results of this experiment are compatible with those of previous studies, indicating that the bacteria and CAZymes mentioned above have important roles in feed degradation. Regression analysis of species and function revealed a significant correlation between function and microbial community species composition (R^2^ = 0.9953, *p* < 0.01). Further, correlation analysis between species and function showed that some species correlated with multiple functions, indicating that changes in microbial community composition will change the overall functional composition [51]; however, molecular techniques can identify types of microbial communities and predict their function, but culturing is a better option for obtaining a precise understanding of microbes. Future work should combine these two methods to elucidate the complexities of microbial community functions in the process of roughage degradation. Peanut vines are susceptible to aflatoxin contamination, and microbial treatment methods have advantages in improving the nutritional value of peanut vine and reducing toxins, with broad prospects. Perhaps the microorganisms screened in this study can be used to enhance the nutritional value of peanut vine or reduce toxins, which is worth paying attention to.

## 5. Conclusions

In summary, this study investigated the attachment characteristics and function of microorganisms involved in peanut vine degradation in the rumen. Our results demonstrate that the peanut vine degraded rapidly from 2 to 24 h, slowing from 24 to 72 h, and the colonization of peanut vine by microorganisms is dynamic in the rumen. Firmicutes and Bacteroidetes were the major sources of CAZymes and probably the primary degraders of plant biomass in the rumen. Researchers can isolate and cultivate these microorganisms or use heterologous expression to produce these enzymes to improve the utilization of crop residues in ruminants.

## Figures and Tables

**Table 1:** The DM, NDF, ADF degradation parameters of peanut vine in rumen of cows.

Index	DM	NDF	ADF
rapid degradation fraction a (%)	18.26 ± 0.96	2.33 ± 0.36	1.18 ± 0.15
slow degradation fraction b (%)	36.27 ± 1.81	32.51 ± 2.27	34.32 ± 2.07
degradation rate constant of slow degradation fraction c (%/h)	0.08 ± 0.02	0.06 ± 0.01	0.06 ± 0.01
effective degradation rate ED (%)	43.51 ± 1.16	22.90 ± 0.90	22.89 ± 0.79

**Table 2:** Alpha diversity indices of microbiota communities attached to peanut vine after incubation in the cow rumen for 0.5, 2, 6, 12, 24, 48, and 72 h.

Alpha Diversity	0.5 h	2 h	6 h	12 h	24 h	48 h	72 h	SEM	*p*-Values
Sobs	1687 ^ab^	1713 ^a^	1658 ^bc^	1621 ^c^	1635 ^c^	1544 ^d^	1516 ^d^	23.27	<0.001
Shannon	6.04 ^ab^	6.12 ^a^	6.04 ^ab^	5.91 ^c^	6.01 ^b^	5.76 ^d^	5.76 ^d^	0.047	<0.001
Simpson	0.008 ^b^	0.008 ^b^	0.009 ^b^	0.012 ^a^	0.009 ^b^	0.013 ^a^	0.013 ^a^	0.001	<0.001
Ace	2153 ^a^	2173 ^a^	2093 ^ab^	2042 ^bc^	2019 ^bcd^	1991 ^cd^	1935 ^d^	40.81	<0.001
Chao1	2170 ^ab^	2198 ^a^	2138 ^abc^	2087 ^bcd^	2059 ^cd^	1994 ^d^	1988 ^d^	48.80	<0.001
Coverage	0.975	0.975	0.976	0.976	0.977	0.977	0.977		

Different letters (a–d) indicate significant differences within the same row (*p* < 0.05); SEM, standard error of mean.

## Data Availability

The original contributions presented in the study can be contacted to the corresponding author.

## Supplementary Materials

The following supporting information can be downloaded at: https://www.mdpi.com/article/10.3390/life14070802/s1, Figure S1: Phylum level relative abundance analysis of microbiota communities attached to peanut vine by Metagenomics. Figure S2: Genus level relative abundance analysis of microbiota communities attached to peanut vine by Metagenomics. Figure S3: Principal coordinate analysis of class and family level CAZyme diversity and analysis of similarities among groups. Figure S4: Species (top 20) and function (families level) contribution analysis. Figure S5: Association and model predictive analysis.
