# Supplementary material for: Integrated Multi-Omics Reveals New Ruminal Microbial Features Associated with Peanut Vine Efficiency in Dairy Cattle
Source: Life (Basel). 2024 Jun 26;14(7):802. doi: 10.3390/life14070802 (PMC11277927; doi:10.3390/life14070802)
Supplement: Supplementary file 1 [file life-14-00802-s001.zip › life-2998231-supplementary.pdf]

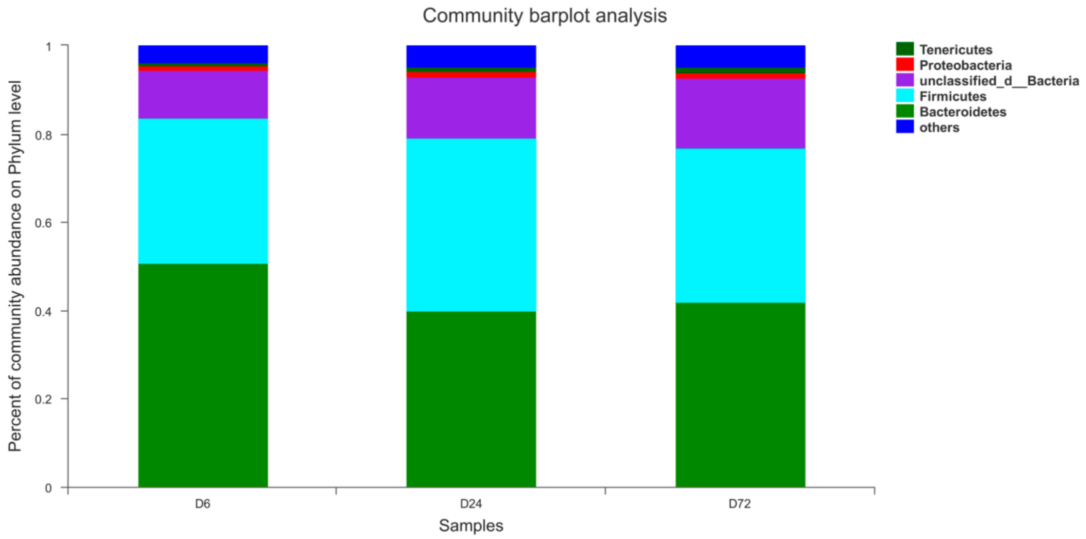

Supplementary Figure S1. Phylum level relative abundance analysis of microbiota communities attached to peanut vine after incubation for 6, 24, and 72 h in cow rumen based on metagenome shotgun sequencing. D6, D24, and D72 represent peanut vine samples incubated in cow rumen for 6, 24, and 72 h, respectively. Phyla with relative abundance < 0.01 in all samples were classified as others.

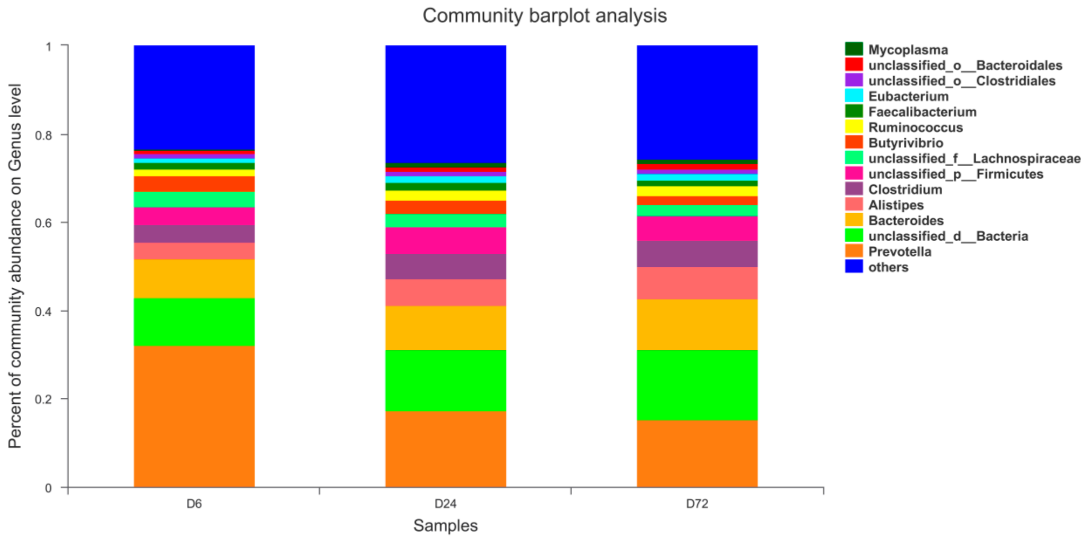

Supplementary Figure S2. Genus level relative abundance analysis of microbiota communities attached to peanut vine after incubation for 6, 24, and 72 h in cow rumen, based on metagenome shotgun sequencing. D6, D24, and D72 represent peanut vine samples incubated in cow rumen for 6, 24, and 72 h, respectively. Genera with relative abundance < 0.01 in all samples were classified as others.

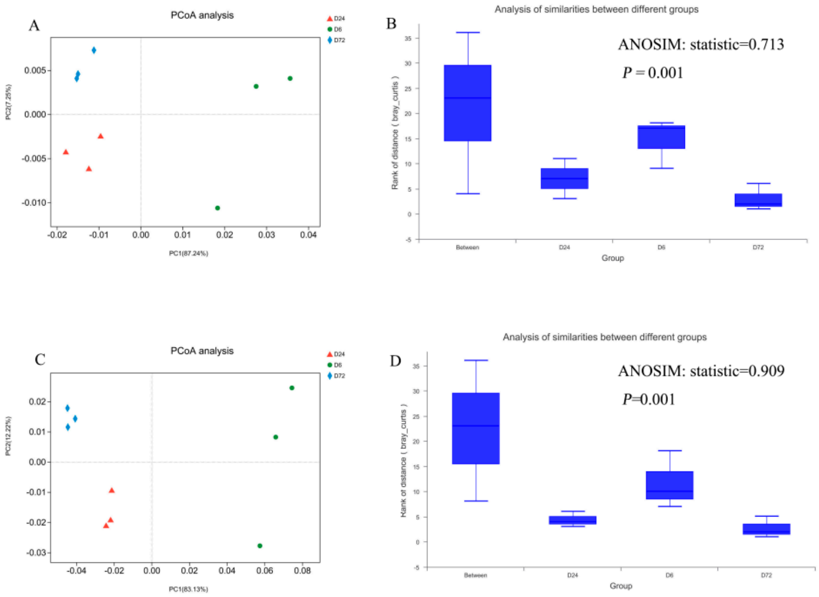

Supplementary Figure S3. (A, C) Principal coordinate analysis (PCoA) of class and family level CAZyme diversity. (B, D) Analysis of Similarities among the 6, 24, and 72 h groups using the Bray-Curtis metric. ANOSIM analysis showed significant differences among the three groups ($p < 0.05$). D6, D24, and D72 represent peanut vine samples incubated in cow rumen for 6, 24, and 72 h, respectively.

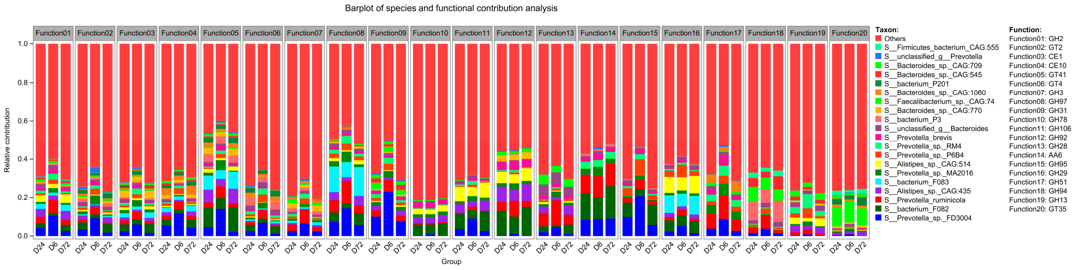

Supplementary Figure S4. Species (top 20) and function (families level) contribution analysis. The graphics illustrate the relative abundance of a taxa participating in a function, calculated by summing the abundance of a taxon participating in a pathway assigned to the total abundance of all taxa involved in the function. D6, D24, and D72 represent peanut vine samples incubated in cow rumen for 6, 24, and 72 h, respectively.

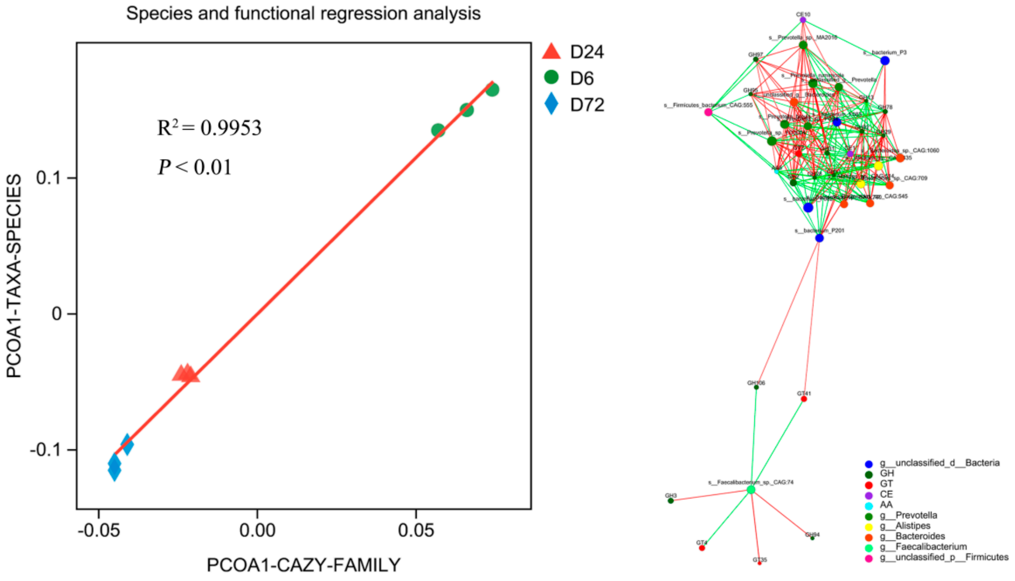

(a) (b)

Supplementary Figure S5. Association and model predictive analysis. (a) Species and functional regression analysis. The abscissa represents the beta diversity index of function, and the ordinate the beta diversity index of species. $R^2 = 0.9953$, indicating that the diversity of community species and function β is very similar. (b) Correlation analysis between species and functions. Colors indicate bacteria (phylum level) and function colors (class level). Line color represents correlation: red, positive correlation; green, negative correlation. The thicker the line, the higher the correlation; the more lines, the stronger the correlation. D6, D24, and D72 represent peanut vine samples incubated in cow rumen for 6, 24, and 72 h, respectively.
